# Unchanged Early Diffusion Tensor Imaging Along Perivascular Space Index After Amyloid‐Targeting Disease‐Modifying Therapy in Alzheimer's Disease: A Preliminary Study

**DOI:** 10.1002/jmri.70118

**Published:** 2025-09-08

**Authors:** Tatsushi Oura, Hiroyuki Tatekawa, Akitoshi Takeda, Ayako Omori, Natsuko Atsukawa, Shu Matsushita, Daisuke Horiuchi, Hirotaka Takita, Taro Shimono, Daiju Ueda, Yoshiaki Itoh, Yukio Miki

**Affiliations:** ^1^ Department of Diagnostic and Interventional Radiology, Graduate School of Medicine Osaka Metropolitan University Osaka Japan; ^2^ Department of Neurology, Graduate School of Medicine Osaka Metropolitan University Osaka Japan; ^3^ Department of Artificial Intelligence, Graduate School of Medicine Osaka Metropolitan University Osaka Japan

**Keywords:** Alzheimer's disease, disease‐modifying therapy, DTI‐ALPS index, glymphatic function, lecanemab

Alzheimer's disease (AD) is characterized by the progressive accumulation of amyloid‐β peptides in the brain parenchyma, and impairment of interstitial waste clearance via the glymphatic system is suggested as one contributing factor [1, 2]. Recently approved disease‐modifying (DM) monoclonal antibodies, such as lecanemab, are expected to slow cognitive decline by improving amyloid β clearance [3, 4, 5]. Diffusion tensor imaging along the perivascular space (DTIALPS) index has emerged as a noninvasive surrogate marker suggested to be associated with glymphatic activity [6]. This index declines with normal aging and is significantly lower in patients with AD than in cognitively normal individuals [6, 7, 8]. Although DM therapy (DMT) may affect the DTI‐ALPS index, no studies have examined longitudinal changes in the DTI‐ALPS index following DMT initiation. Therefore, this study quantified the DTI‐ALPS index in patients with AD before and 3 months after the initiation of amyloid‐targeting DMT to generate provisional reference values for the longitudinal assessment of future DM‐treated cohorts.

This investigation comprised a two‐step observational analysis. (1) From a publicly available Open Access Series of Imaging Studies (OASIS) dataset (http://oasis‐brains.org/), cognitively normal volunteers (23 paired data) who underwent two identical DTI acquisitions were used solely to characterize within‐participant variability and to derive the sample size requirement and associated 90% confidence interval (CI) limits for the subsequent equivalence test [9]. (2) Paired DTI‐ALPS indices at baseline and 3 months after the initiation of DMT were evaluated at our institution.

Participants were prospectively and consecutively recruited between December 1, 2023, and February 28, 2025. Included participants were: (i) diagnosed with AD by neurologists; (ii) underwent brain magnetic resonance imaging (MRI) as part of screening for DMT and subsequently initiated lecanemab therapy. Only participants who provided written informed consent were included.

All DTI data were obtained from a 3T MRI system, Magnetom Vida (Siemens Healthineers, Erlangen, Germany; 20 channel head coil), with an identical singleshot echoplanar DTI protocol (*b* = 1000 s/mm^2^, 30 directions with one *b*0 volume). Baseline MRI was performed within 3 months before the first lecanemab dose, and follow‐up MRI was performed exactly 3 months after treatment initiation.

Before analysis, every DTI data was cleaned to reduce random noise, stripped of Gibbs ringing artifacts, corrected for motion and eddy current‐related distortions, and adjusted for intensity bias using MRtrix3 (version 3.0.4; https://www.mrtrix.org). Subsequently, the functions VECREG, FLIRT, and FNIRT, provided by the FMRIB software library (FSL, version 6.0.7.3; https://www.fmrib.ox.ac.uk/fsl), were used for DTI vector reorientation, linear, and non‐linear registration, respectively, using an automated calculation method of DTI‐ALPS index [8]. Mean diffusivities along Dxx, Dyy, and Dzz within ROIs of projection and association fibers from bilateral hemispheres were averaged, yielding a single DTI‐ALPS index. The detailed processing overview and script are presented in Figure 1 and Data S1, respectively.

**FIGURE 1:**
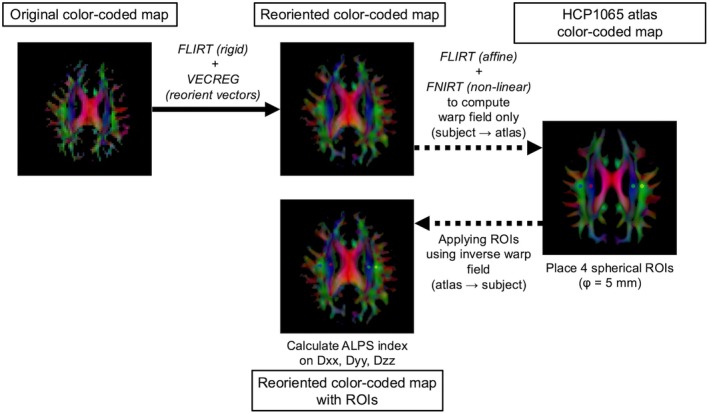
Workflow for automated DTI‐ALPS index calculation. DTI data are visualized as color‐coded direction maps for illustration purposes. (1) FA maps are rigidly aligned to the HCP1065 FA atlas using FLIRT, and tensor orientations are applied using VECREG to yield re‐oriented maps (center‐top). (2) The re‐oriented FA maps are further aligned to the HCP1065 FA atlas with affine + non‐linear registration (FLIRT → FNIRT) to generate a subject‐to‐atlas warp field (right) with the purpose of generating the warp field required for subsequent inverse mapping. (3) The HCP1065 atlas provides color‐coded direction maps and other DTI‐derived maps, thereby facilitating an intuitive definition of the region of interest (ROI). In the atlas space, four spherical ROIs (diameter = 5 mm) are placed on the bilateral projection (blue) and association (green) fibers. (4) Using the inverse warp, these ROIs are mapped back to the re‐oriented subject space and overlaid on the directional map (center‐bottom). While the ROIs are visually inspected by two radiologists, no manual adjustment of the ROI placement is required. Finally, the mean diffusivities along Dxx, Dyy, and Dzz within bilateral ROIs are then extracted to compute the DTI‐ALPS index.

Repeated DTI‐ALPS indices calculation of 23 OASIS volunteers yielded a within‐participant standard deviation (SD) of 0.07. The data were used to characterize variability for sample‐size planning and equivalence margins, not for cross‐protocol comparisons. Setting an equivalence margin of *δ* = ±0.05 (≈0.7 SD) as clinically negligible for therapy‐related changes, a two one‐sided test (TOST; one‐sided *α* = 0.05, power = 0.80) indicated that 13 paired participants would be sufficient. The 90% CI is the interval that corresponds to these dual *α* = 0.05 tests and is therefore standard in equivalence studies. The detailed volunteer data are shown as Supplement data. Then, baseline and three‐month participants' DTI‐ALPS indices were analyzed with the same TOST, declaring equivalence when the entire 90% CI lay within ±0.05 (both *p* < 0.05); a paired two‐tailed *t*‐test served as a secondary check.

Of 32 DMT recipients with AD, 13 met the inclusion criteria and satisfied power requirements. The mean age was 72 years (5 men and 8 women).

Mean DTI‐ALPS index was 1.515 ± 0.152 at baseline and 1.513 ± 0.161 at 3 months. The change (*δ* = 0.002) had a 90% CI of −0.049 to +0.045, entirely within the ±0.05 equivalence margin; both one‐sided TOST *p* values were < 0.05 (*p*_1_ = 0.035, *p*_2_ = 0.047), establishing equivalence. This constrains any mean 3‐month effect to ≤ 0.049 under our protocol. A paired *t*‐test showed no significant difference (*t*_12_ = 0.08, *p* = 0.94), confirming no 3‐month changes of DTI‐ALPS index (Figure 2).

**FIGURE 2:**
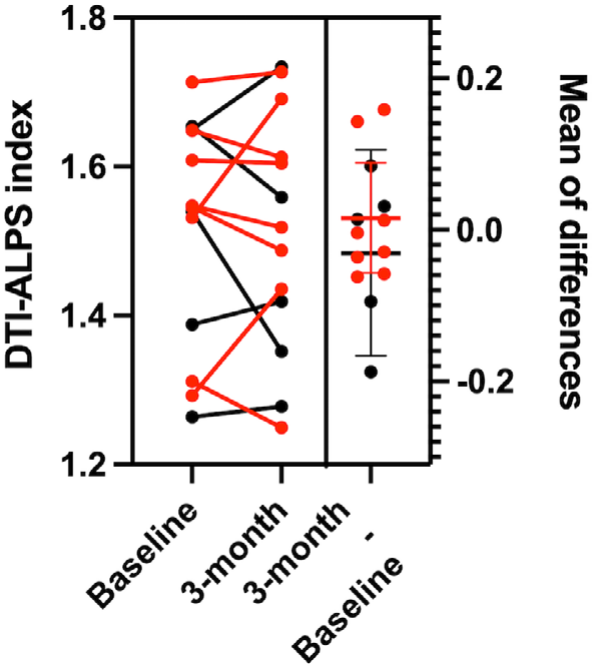
Dynamic change of DTI‐ALPS index for pre‐ and post‐DMT. Each dot represents one participant; lines connect paired baseline and 3‐month values. Individual data points are color‐coded by sex (red: females, black: males). The paired *t*‐test shows no statistically significant differences between pre‐ and post‐DMT (*p* = 0.94). Note, one participant developed the amyloid‐related imaging abnormalities effusion (ARIA‐E) at 3 months; the DTI‐ALPS index was 1.714 at baseline and 1.727 at 3 months.

In this small but power‐estimated cohort, the DTI‐ALPS index showed no measurable improvement during the first 3 months of DMT initiation. Despite the small sample size, the findings can provide an initial benchmark for interpreting the DTI‐ALPS index in future studies evaluating the effect of DMT for AD.

The absence of early DTI‐ALPS index improvement suggests that even though DMT reduces plaque burden, the diffusion properties of perivascular spaces measured by DTI‐ALPS do not change in the short term. DMT can reduce plaque burden and slow further cognitive worsening but does not restore lost function, likely reflecting the fact that neuronal damage and clearance system deficits have already been well established [3, 5]. Such observations, therefore, may reflect a multifactorial disease process that is not rapidly reversible during symptomatic stages, resulting in an unchanged early DTI‐ALPS index.

This study had some limitations. First, the sample size was small, which prevented reliable subgroup or multivariate analyses. Furthermore, since only 3‐month changes were assessed, longitudinal cohorts may clarify responder versus non‐responder traits. Second, only participants treated with lecanemab were included in this study. As other DMTs, such as donanemab, are also available, future research should compare these agents to determine whether the findings hold across therapies. Third, DTI‐ALPS derived metrics may reflect underlying axonal geometry or anatomical complexities rather than purely glymphatic function [10], which should be considered when interpreting the results. Fourth, because we did not acquire age‐ and sex‐matched controls with two test–retest scans using the same sequence, we could not directly quantify sequence‐specific systematic differences or test–retest variability; this limits the generalizability of our equivalence inference and power analysis.

In conclusion, the DTI‐ALPS index did not change during the first 3 months of lecanemab therapy. However, larger cohorts and longer follow‐up periods are required to clarify the temporal relationship between DTI‐ALPS index dynamics and therapeutic effects.

## Conflicts of Interest

The authors declare no conflicts of interest.

## Supporting information


**Data S1:** jmri70118‐sup‐0001‐Supinfo.docx.
