# Supplementary material for: Unchanged Early Diffusion Tensor Imaging Along Perivascular Space Index After Amyloid‐Targeting Disease‐Modifying Therapy in Alzheimer's Disease: A Preliminary Study
Source: J Magn Reson Imaging. 2025 Sep 8;62(6):1892–4. doi: 10.1002/jmri.70118 (PMC12604541; doi:10.1002/jmri.70118)
Supplement: Supplementary file 1 — Data S1: jmri70118‐sup‐0001‐Supinfo.docx. [file JMRI-62-1892-s001.docx]

| **Supplemental Table 1.** Characteristics and clinical data of the participants | |
| --- | --- |
| Sex (Male/Female) | 5/8 |
| Age (years, mean with range) | 72 (56–82) |
| MMSE at baseline (mean with range) | 24.6 (22–28) |
| ARIA-E (edema) at 3 months post-initiation (n) | 1 |
| ARIA-H (microbleeds) at 3 months post-initiation (n) | 0 |
| Interval between DTI scans (days, mean with range) | 113 (90-165) |

| **Supplemental Table 2.** DTI-ALPS index for normal control of OASIS dataset | |
| --- | --- |
| Age (years) | 66 ± 16.9 |
| Sex (Females/males) | 10/13 |
| MMSE | 29.64 ± 0.79 |
| Interval (days) | 709 ± 328 |
| Test | 1.567 ± 0.184 |
| Retest | 1.585 ± 0.201 |
| Difference | -0.018 ± 0.07 |
| Values are presented as mean ± standard deviation, except for Sex, which is shown as number of females/males. | |

**DTI parameters of Our institution and OASIS dataset**

All MRI data of our institution were acquired using a 3 Tesla scanner, Magnetom Vida (Siemens Healthineers, Erlangen, Germany; 20 channel head coil), with an identical single shot echo planar DTI protocol (TR/TE = 4500/81 ms; flip angle = 90°; voxel size = 2 × 2 × 2 mm³; *b* = 1000 s/mm², 30 directions with one *b0* volume).

All MRI data from the OASIS-3 database were acquired using a 3 Tesla scanner (Siemens Healthineers, Erlangen, Germany) with an identical single‑shot echo‑planar DTI protocol (TR/TE = 11000/87 ms; flip angle = 90°; voxel size = 2.5 × 2.5 × 2.5 mm³; *b* = 1000 s/mm², 64 directions with one *b0* volume). Detailed information is available on the OASIS website.

#!/bin/bash

## Please prepare dti.bval, dti.bvec, dti.json, dti.nii.gz files

## Please create acqparams.txt and index.txt

###############################################################################

# WARNING

# -------

# 1. As of FSL 6.0.7, the utility ‘vecreg’ is no longer included in the official

# FSL distribution. Install it manually or replace it with an equivalent

# tool (e.g., ANTs ReorientTensorImage or MRtrix3 transformcalc) before

# running this pipeline.

#

# 2. Ensure that the environment variable $FSLDIR is exported

# and point to your FSL installation directory.

# The script will abort if it is undefined.

###############################################################################

#########################################

## Step 1: Brain extraction from b=0

#########################################

# Extract the first b=0 volume

#fslroi dti.nii.gz b0 0 1

# Brain extraction to create mask

#bet b0 b0_brain -m -R -f 0.25 -g 0.05 # Generates b0_brain_mask.nii.gz

#########################################

## Step 2: Eddy current correction

#########################################

#eddy \

# --imain=dti.nii.gz \

# --mask=b0_brain_mask.nii.gz \

# --acqp=acqparams.txt \

# --index=index.txt \

# --bvecs=dti.bvec \

# --bvals=dti.bval \

# --out=dti_eddy_corrected \

# --slm=linear

#########################################

## Step 3: Tensor fitting and FA map generation

#########################################

#dtifit \

# --data=dti_eddy_corrected.nii.gz \

# --out=dti \

# --mask=b0_brain_mask.nii.gz \

# --bvecs=dti_eddy_corrected.eddy_rotated_bvecs \

# --bvals=dti.bval \

# --save_tensor

#########################################

## When use MRtrix3

#########################################

## Step 1-2: Prepare preprocessed images (existing code reused)

mrconvert dti.nii.gz dti.mif -fslgrad dti.bvec dti.bval -datatype float32 # MRtrix3

dwidenoise dti.mif dti_den.mif -noise dti_noise.mif # MRtrix3

mrdegibbs dti_den.mif dti_den_unr.mif -axes 0,1 # MRtrix3

dwifslpreproc dti_den_unr.mif dti_den_unr_preproc.mif -pe_dir AP -rpe_none -eddy_options "--slm=linear" # MRtrix3

dwibiascorrect ants dti_den_unr_preproc.mif dti_den_unr_preproc_unbiased.mif -bias bias.mif # MRtrix3

dwi2mask dti_den_unr_preproc_unbiased.mif mask_den_unr_preproc_unbiased.nii.gz # MRtrix3

mrconvert dti_den_unr_preproc_unbiased.mif dti_den_unr_preproc_unbiased.nii.gz -export_grad_fsl dti_den_unr_preproc_unbiased.bvec dti_den_unr_preproc_unbiased.bval # MRtrix3

## Step 3: Create diffusion tensor and maps

# Generate tensor and diffusion metrics

dtifit --bvals=dti_den_unr_preproc_unbiased.bval --bvecs=dti_den_unr_preproc_unbiased.bvec --data=dti_den_unr_preproc_unbiased.nii.gz --mask=mask_den_unr_preproc_unbiased.nii.gz --out=dti --save_tensor # FSL

#########################################

## Step 4: Linear registration to FSL_HCP1065_FA_1mm

#########################################

ATLAS_FA="$FSLDIR/data/standard/FSL_HCP1065_FA_1mm.nii.gz"

# Rigid-body (6 DOF) alignment of FA map to the standard atlas

flirt -ref $ATLAS_FA -in dti_FA.nii.gz -omat fa2HCP1065_rigid.mat -dof 6 \

-searchrx -90 90 -searchry -90 90 -searchrz -90 90

# Apply linear transformation to FA map

flirt -ref $ATLAS_FA -in dti_FA.nii.gz -applyxfm -init fa2HCP1065_rigid.mat -out dti_FA_ATLAS.nii.gz

#########################################

## Step 5: Tensor and eigenvector reorientation using vecreg

#########################################

# Requires vecreg to be installed separately (part of FSL or external)

# Reorient tensor image using linear transform

vecreg -i dti_tensor.nii.gz -o dti_ATLAS_tensor.nii.gz -r $ATLAS_FA -t fa2HCP1065_rigid.mat

# Reorient principal eigenvector image (V1)

vecreg -i dti_V1.nii.gz -o dti_ATLAS_V1.nii.gz -r $ATLAS_FA -t fa2HCP1065_rigid.mat

#########################################

## Step 6: Extract individual tensor components and visualize directionality

#########################################

# Extract transformed tensor components from reoriented tensor image

# Dxx (component 0)

#fslroi dti_ATLAS_tensor.nii.gz dti_ATLAS_Dxx.nii.gz 0 1

# Dyy (component 3)

#fslroi dti_ATLAS_tensor.nii.gz dti_ATLAS_Dyy.nii.gz 3 1

# Dzz (component 5)

#fslroi dti_ATLAS_tensor.nii.gz dti_ATLAS_Dzz.nii.gz 5 1

# Create a directionally encoded color FA (DEC-FA) image by multiplying V1 with FA

#fslmaths dti_ATLAS_V1.nii.gz -mul dti_FA_ATLAS.nii.gz dti_ATLAS_DEC.nii.gz

# Visualize the DEC-FA image overlaid on FA using fsleyes

# afni can also create Color maps

3dcalc -prefix DTIout_ATLAS.nii.gz -a 'dti_ATLAS_V1.nii.gz[0..2]' -c 'dti_FA_ATLAS.nii.gz' -expr 'c*step(c-0.2)*255*abs(a)'

3dThreetoRGB -prefix DTI_ATLAS_ColorMap.nii.gz -anat DTIout_ATLAS.nii.gz

#########################################

## Step 7: Nonlinear registration to standard space

#########################################

flirt -in dti_FA_ATLAS.nii.gz \

-ref $ATLAS_FA \

-omat fa2HCP1065_affine.mat \

-out dti_FA_flirt.nii.gz

fnirt --in=dti_FA_ATLAS.nii.gz \

--aff=fa2HCP1065_affine.mat \

--ref=$ATLAS_FA \

--iout=dti_FA_warped_to_HCP1065.nii.gz \

--cout=dti_to_HCP1065_warp.nii.gz \

--config=$FSLDIR/etc/flirtsch/FA_2_FMRIB58_1mm.cnf

#########################################

## Step 8: Inverse warp to transform ROI from standard to native space

#########################################

invwarp --warp=dti_to_HCP1065_warp.nii.gz \

--ref=dti_FA_ATLAS.nii.gz \

--out=HCP1065_to_dti_warp.nii.gz

# ROI label definitions:

# Please change the path of ROI data (FSL_HCP1065_FA_ROI.nii.gz)

# 1 = right CR, 2 = left CR, 3 = right SLF, 4 = left SLF

ATLAS_ROI=Path/to/FSL_HCP1065_FA_ROI.nii.gz # Please define it

applywarp --in=$ATLAS_ROI \

--ref=dti_FA_ATLAS.nii.gz \

--warp=HCP1065_to_dti_warp.nii.gz \

--interp=nn \

--out=ROI_in_dti_FA_ATLAS_space.nii.gz

#########################################

# Please review whether ROI data (ROI_in_dti_FA_ATLAS_space.nii.gz)

# is appropriately located in the DTI direction map (dti_FA_ATLAS.nii.gz dti_ATLAS_DEC.nii.gz or DTI_ATLAS_ColorMap.nii.gz).

# Then, please calculate ALPS index from dti_ATLAS_Dxx.nii.gz, dti_ATLAS_Dyy.nii.gz, and dti_ATLAS_Dzz.nii.gz

# or dti_ATLAS_V1.nii.gz using ROI_in_dti_FA_ATLAS_space.nii.gz

#########################################
